# Prospective Observational Study of Single-Site Multiport Per-umbilical Laparoscopic Endosurgery versus Conventional Multiport Laparoscopic Cholecystectomy: Critical Appraisal of a Unique Umbilical Approach

**DOI:** 10.1155/2014/909321

**Published:** 2014-04-30

**Authors:** Priyadarshan Anand Jategaonkar, Sudeep Pradeep Yadav

**Affiliations:** ^1^Department of General & Laparoscopic Surgery, Mahatma Gandhi Institute of Medical Sciences, Sevagram, Wardha, Maharashtra 442102, India; ^2^Department of General & Laparoscopic Surgery, Jagjivanram Western Railway Hospital, Mumbai Central, Mumbai, Maharashtra 400008, India

## Abstract

*Purpose*. This prospective observational study compares an innovative approach of Single-Site Multi-Port Per-umbilical Laparoscopic Endo-surgery (SSMPPLE) cholecystectomy with the gold standard—Conventional Multi-port Laparoscopic Cholecystectomy (CMLC)—to assess the feasibility and efficacy of the former. *Methods*. In all, 646 patients were studied. SSMPPLE cholecystectomy utilized three ports inserted through three independent mini-incisions at the umbilicus. Only the day-to-day rigid laparoscopic instruments were used in all cases. The SSMPPLE cholecystectomy group had 320 patients and the CMLC group had 326 patients. The outcomes were statistically compared. * Results*. SSMPPLE cholecystectomy had average operative time of 43.8 min and blood loss of 9.4 mL. Their duration of hospitalization was 1.3 days (range, 1–5). Six patients (1.9%) of this group were converted to CMLC. Eleven patients had controlled gallbladder perforations at dissection. The Visual Analogue Scores for pain on postoperative days 0 and 7, the operative time, and the scar grades were significantly better for SSMPPLE than CMLC. However, umbilical sepsis and seroma outcomes were similar. We had no bile-duct injuries or port-site hernias in this study. * Conclusion*. SSMPPLE cholecystectomy approach complies with the principles of laparoscopic triangulation; it seems feasible and safe method of minimally invasive cholecystectomy. Overall, it has a potential to emerge as an economically viable alternative to single-port surgery.

## 1. Introduction 

*C*onventional* M*ulti-port* L*aparoscopic* C*holecystectomy (CMLC) is the gold-standard for tackling benign gallbladder diseases; it generally requires 4 (sometimes even 5 or more) ports spread across different quadrants of abdomen. Recently, the surgeons' quest for reducing the access-trauma by reducing the number of ports has led to several technical modifications regarding minimally invasive cholecystectomy [1, 2]. And the natural-orifice transluminal endoscopic surgery (NOTES) with its potential to achieve completely scarless abdomen, though the most sought for, seems to have fallen out of favor owing to the technical complexity, the prolonged learning curve, and the questionable safety due to the issues regarding closure of mucosal breach. Logically, the per-umbilical approach, with its potential to produce almost the similar results, has been warmly welcomed by the surgeons and the industry. However, this “third generation” surgery is far from being accepted as the standardized approach due to the lack of ease and uniformity in instrumentation/technique apart from the paucity of convincing data. In this paper, we present an investigational technique—what we called it as the* S*ingle-*S*ite* M*ulti-*P*ort* P*er-umbilical* L*aparoscopic* E*ndo-surgery (SSMPPLE). We further compare it prospectively with CMLC for its critical appraisal. The encouraging results of our first 15 patients (10 straightforward cases and 5 acute cholecystitis cases) prompted us to undertake this comparative analysis. These patients have been excluded from this study. As such, SSMPPLE should be considered distinct methodology from the conventional single-incision technique.

## 2. Materials and Methods 

### 2.1. Informed Consent

One-to-one discussion sessions were arranged between each of the 646 patients and the surgeon to converse about the technical details of procedure. The investigative nature of the procedure along with its likely advantages and disadvantages/complications at the backdrop of the well-established technique of CMLC were clearly explained to all; subsequently, they were allowed to choose one of the operative techniques. Accordingly, a written informed consent was obtained from everybody.

### 2.2. Study Population

Following criteria were designed for including or excluding the subjects for this study.

### 2.3. Inclusion Criteria

The inclusion criteria of this study comprised of: (1) biliary colic, (2) chronic calculus cholecystitis, (3) acute calculus cholecystitis, (4) gallbladder polyps with cholelithiasis, (5) gallbladder mucocele, (6) gallbladder empyema, and (7) biliary pancreatitis.

### 2.4. Exclusion Criteria

Anticipating the technical difficulty, we offered upfront CMLC or open cholecystectomy to the following patients. Hence, these patients were excluded from the study: (1) patients with choledocholithiasis, (2) perforated gallbladder, (3) remnant calculus cholecystitis, (4) Mirizzi syndrome, (5) suspected carcinoma gallbladder, (6) obese patients with the body mass index (BMI) >35 kg/m^2^, and (7) patients unfit for laparoscopy.

### 2.5. Patient Information

#### 2.5.1. SSMPPLE Group

Three hundred and twenty patients underwent SSMPPLE cholecystectomy from March 2007 to March 2011. Out of them, 221 were females and 99 were males. The mean BMI for the males was 27.7 kg/m^2^ (range, 17–31.5) and that for the females was 28.4 kg/m^2^ (range, 19–33.7). The mean age of the males was 42.5 years (range, 17–64) and that for the females was 45.3 years (range, 22–68). Eighty patients in this series had some form of medical comorbidity. Indications of surgery included both “simple” as well as “difficult” gallbladder pathologies. We could also successfully apply SSMPPLE technique to patients with abdominal scars of prior surgeries like laparoscopic tubal ligation (*n* = 22) with umbilical scar, laparoscopic appendectomy (*n* = 6), and midline laparotomy (*n* = 6) (Table 1).

#### 2.5.2. CMLC Group

Out of a total of 326 patients operated during the same time frame, 95 were males and 231 were females. The mean BMI for males was 25.3 kg/m^2^ (range, 18–30) and that for the females was 27.5 kg/m^2^ (range, 18–32.3). Eighty eight patients had some form of medical comorbidity. As with SSMPPLE group, this arm also included similar varieties of straightforward as well as technically difficult cases. Five patients had midline scar of exploratory laparotomy, 25 patients had umbilical scar of laparoscopic tubal ligation, and 4 patients had port scars of laparoscopic appendectomy (Table 1).

### 2.6. Preoperative Assessment and Preparation

We evaluated all these 646 patients preoperatively by the same biochemical (complete blood count, liver, and renal function tests) and the radiological (abdominal ultrasonography) tests. The decision to offer contrast-enhanced abdominal computed tomography scans or magnetic resonance cholangiography for studying biliary system in detail was taken on case-to-case basis. However, none of the patients from either group needed these special tests. Preanesthesia check was obtained for their fitness to withstand general anesthesia. Preoperative optimization was ensured for all patients from both groups, especially for smokers (by abstinence from smoking) and cardiac patients (by enhancing the exercise tolerance). We do not perform per-operative cholangiography routinely. In an attempt to keep a check on the rate of umbilical sepsis, all patients were subjected to meticulous umbilical cleaning preoperatively (twice the previous evening and once on the day of surgery) with chlorhexidine.

### 2.7. Instrumentation

Only “day-to-day” autoclavable laparoscopic instruments were used in this study. For SSMPPLE cholecystectomy, we used one 10 mm trocar (for 30° 10 mm laparoscope) and two 5 mm valved threaded plastic trocars (for right and left hand working instruments). Monopolar electrosurgery was used for majority of the cases. Except for the Harmonic scalpel (Ethicon Endosurgery, Cincinnati, OH, USA) engaged selectively for the technically difficult cases (17 from SSMPPLE group and 15 from CMLC group), no other specialized equipment was used. Standard port-closure needle was used for the gallbladder traction when required. Though not used in this series, it would be advisable to use extralong instruments if available.

### 2.8. Surgical Team

To avoid bias, all the patients of both groups were operated on by the same surgeon and the surgical team.

### 2.9. Anesthesia and Patient Position

All the patients of both groups were operated under general anesthesia. They were placed in supine position with 30° head-up and 20° right-up position. A nasogastric tube was inserted and single-dose of broad-spectrum antibiotic was administered at induction in all. The monitor was placed at the right shoulder of the patient. The surgeon stood on the left of the patient and the camera assistant stood on the left of the surgeon.

### 2.10. Clinical Parameters Studied

Postoperative outcomes studied for both groups were operative time (defined as the time interval between the first port entry till the last port closure), blood loss, bile duct injury, viscus injury, gallbladder perforation during dissection, conversion to either CMLC or open, postoperative pain, stages of recovery, duration of hospitalization, umbilical seroma/sepsis, cosmetic results, and the rate of port-site hernia. The Visual Analogue Scale (VAS, 0–10) was used for assessing the postoperative pain on days 0, 1, 7, and 30. Considering the suboptimal educational and socioeconomic background of our rural patients, we developed an easy-to-use scar grading scale (I-Thrilled, II-Happy, III-Not bothered, and IV-Unhappy) as per the subjective feeling about the scar they have received. We felt this scale was just the handy method of judging the scar outcomes in our part of the world. Although this system lacked the detailed questionnaire (and hence detailed objective evaluation) regarding the cosmetic outcomes, it assessed the cosmetic results on a gross scale.

### 2.11. Statistical Analysis

Using SPSS 10.0 software (SPSS Inc., Chicago, IL, USA), Pearson's Chi-square test was applied to assess the statistically significant difference between the variables. This difference was considered significant if the *P* value was <0.05.

### 2.12. Surgical Techniques 

#### 2.12.1. SSMPPLE Cholecystectomy

**Figure 1:**
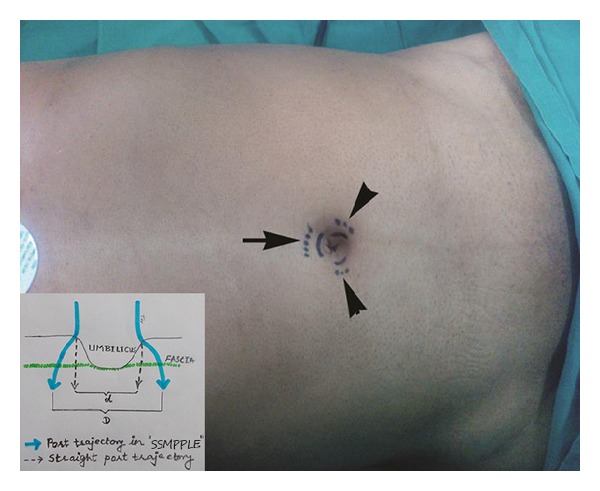
Incisions for port placement. Solid lines indicate the skin incisions and dotted lines indicate the fascial trajectories. This resulted in spacing the trocars away.* Inset.* Diagrammatic representation of the ports pathways. Note that the intertrocar distance is more with curved paths (D) than with straight (d).

**Figure 2:**
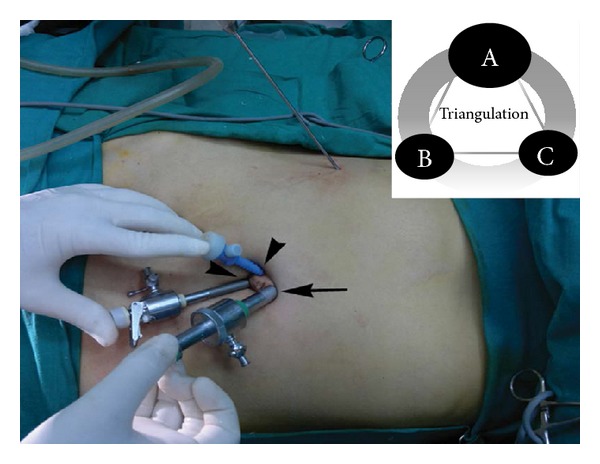
Port position. One 10 mm (arrow) and two 5 mm (arrow heads) ports placed* on *the umbilical mound in triangular fashion (*Upper inset)*. Note the port-closure needle at the right hypochondrium for gallbladder traction.

The technique of creation of pneumoperitoneum by Veress needle was subject to the shape of umbilicus and the presence of abdominal scar (if any) of previous surgery. In the patient with wide umbilicus (defined as ≥ 2.5 cm diameter) and without any abdominal scar, a 2 mm stab incision was placed at the 12 O'clock position* on/just inside* the umbilical mound for inserting the Veress needle before creating the pneumoperitoneum. In these patients, we set the intra-abdominal pressure (IAP) at 14 mm Hg. For patients with cardiac and pulmonary comorbidities, we lowered the IAP to 10–12 mm Hg to minimize the detrimental effects of the raised IAP. The pediatric patients were set on 8–10 mm Hg of IAP. The stab incision was then converted into 11 mm curvilinear skin-crease incision (in line with the umbilical mound) through which a 10 mm sharp trocar was introduced. This was used for 10 mm 30° laparoscope. Two 5 mm, one at the 8 O'clock for the left-hand-working instrument and the another at the 4 O'clock position for the right-hand-working instrument were introduced through the similar 5 mm curvilinear skin-crease incisions* on/just inside* the umbilical mound to achieve the triangular trocar ergonomics. The fascial trajectories for all these three trocars were angled centrifugally by 3-4 mm from the respective cutaneous entries (Figures 1 and 2). This modification helped in reducing the intracorporeal “sword-fighting” of the instruments. Moreover, the obliquity of the trocar paths tends to act as a “flap-valve” mechanism in preventing the trocar-site herniation postoperatively.

However, this assembly of 12 O'clock (10 mm)–4 O'clock (5 mm)–8 O'clock (5 mm) can be changed to 6 O'clock (10 mm)–2 O'clock (5 mm)–10 O'clock (5 mm) depending on surgical team's comfort. After this series, we have used the latter in 17 patients with no added advantage.

The pneumoperitoneum helped in stretching the umbilical ring and, thus, purchased some added distance between the trocars and prevented them falling “on-top” of each other (Figure 2,* inset*). Valves of both the 5 mm trocars were kept outwardly placed—one of them was used for CO_2_ inflow and other one was used for venting the surgical smoke. Alternatively, the CO_2_ cable may be attached to the valve of the 10 mm port. This, along with the light cable, were made to exit from the tops of their respective trocars. Threaded trocars tend to have good grip and prevent gas leak.

Tricks adopted to rectify surgeon-to-camera-assistant collisions and instrument-clashes during the procedure included the following. (1) We adjusted the distant tip of 10 mm cannula to be just inside the peritoneal cavity. This step made it possible to keep the laparoscope withdrawn most of the times, thus, having maximum extracorporeal length of the laparoscope. It could distance the camera-assistant's hand from that of surgeon's. (2) When feasible, extralong laparoscopes were encouraged. (3) Both 5 mm working trocars were inserted 3-4 mm farther into the peritoneal cavity. (4) The camera holding right hand was always laid beneath that of the surgeon's. (5) The surgeon stood on a stool with 0.5 ft height during the whole procedure. This entire surgical assembly gave an adequate “elbow-space” for the operating surgeon as well as the camera-assistant. However, in patients with narrow umbilicus, we preferred to insert all the ports* just outside* umbilical mound to circumvent instrument crowding. Regarding the patients with abdominal scars, anticipating the underlying adhesions in and around the peritoneal side of the umbilicus, we achieved pneumoperitoneum by inserting the Veress needle at the right mid-clavicular line in the right hypochondrium. A miniscope was then inserted through this stab wound and used to visualize the umbilical adhesions if any. Filmy adhesions could be easily swiped with the miniscope itself. In cases of the well-formed adhesions at the umbilicus, instead of using a purely open-laparoscopic technique, a rather safe peritoneal access was achieved by adopting the combination of the “open” laparoscopy (through the curvilinear umbilical incision) counter-monitored by the miniscope via the right hypochondrium.

**Figure 3:**
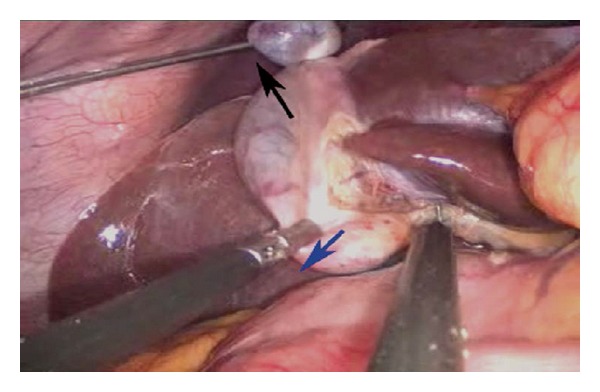
**“**On road” to the critical view of safety. Note the inferolateral traction (blue arrow) by left-hand grasper and cranial traction (black arrow) by the port closure needle to expose the cystohepatic triangle.

The problem of the “floppy” fundus/large gallbladder/bulky liver obliterating the view of the cystohepatic triangle in certain patients was tackled by a simple technique. Commercially available catgut loop was introduced through 5 mm right-hand-working trocar and tightened around the fundus before holding and retracting it cephalad with the standard port-closure needle inserted in the right hypochondrium at the anterior axillary line under the laparoscopic vision. Then, the catgut-loop-tail was held and encircled around the jaws of the port-closure needle in such a way that it locks them and prevents it from slipping during the retraction. This reduced the risk of trauma by its sharp tip (nil in our series). Now, it could be easily maneuvered in any direction as per the requirement of the counter traction. Such a dynamic multidirectional retraction provided by the port-closure actually simulated the 4th port traction of CMLC (Figures 2 and 3) and helped us achieving not only safer but also quicker dissection to accomplish the “critical view of safety” of Strasberg and Soper. Hence, we recommend its liberal use especially for the beginners of the SSMPPLE technique. However, for the thick-walled gallbladders precluding the catgut looping, we performed intracorporeal polypropylene suturing at the fundus before holding and encircling it by the port-closure needle through right hypochondrium in the way described above.

**Figure 4:**
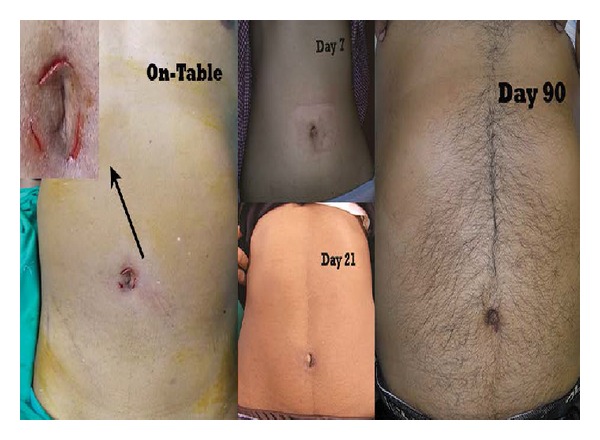
Postoperative scars. Note the undistorted umbilicus with miniscars that are hardly visible.* Inset*. The close-up view of on-table per-umbilical incisions.

The dissection was commenced by retrograde technique by opening the posterior peritoneal leaf at the cystohepatic triangle first followed by the anterior. While the basic principles of the small controlled moves at one time rather than the haphazard ones and dividing the tissues bit-by-bit rather than the “bulk-division” remained the same, we add the following: instead of inserting and advancing both the instruments simultaneously (like one tends to do in the CMLC), introduce the left-hand retracting instrument till the “target organ” and* then* insert the right-hand dissecting instrument to reach the area of interest (and* vice versa* for the left-hand-dominant surgeon). This has helped us in avoiding the intracorporeal instrument-crossing as well as maintaining an optimum distance (that was necessary for the target-organ manipulation) between the tips of these instruments. Once the “critical view of safety” was convincingly achieved, the cystic duct and the artery were doubly clipped with the medium-sized clips by a 5 mm clip-applier inserted through the right-hand-working port before dividing them in between the clips. If deemed necessary, the medium-large clips were used for the wide cystic ducts by inserting a 10 mm clip applier through the 10 mm port. At this time, we exchanged one of the 5 mm working instruments with a 5 mm laparoscope. We transfixed the cystic ducts (22 biliary colic and 5 acute calculus cholecystitis cases) with 2/0 polyglactic acid by the intracorporeal suturing technique in cases where the clip closure was felt insecure. Once dissected completely from its fossa, the gallbladder was extracted in an endobag via the 10 mm port. None of the patient required merging of these three port incisions. Gallstones >1 cm of size (which were likely to obstruct the safe extraction of specimen) were crushed with the stone-holding forceps before removing them piece-meal. Endobags were used for extracting the gallbladders in all cases. Utmost care was exercised to avoid puncturing these endobag. Hemostasis was checked and saline irrigation was given to the gallbladder fossa and the right subdiaphragmatic region for washing out the acidic milieu in an attempt towards reducing the postoperative shoulder pain. We closed* all* three ports in all cases with 2/0 polyglactic acid suture under direct vision. The skin incisions were infiltrated with the mixture of lignocaine and bupivacaine before closing them by 3/0 monofilament absorbable subcuticular sutures. Thus, it was possible to achieve a good cosmetic outcome without distorting the umbilical anatomy after the closure (Figure 4).

#### 2.12.2. Surgical Technique of CMLC

This was in accordance with the standard steps of 4-port laparoscopic cholecystectomy in “American” patient positioning. None of the patients required any extra port. Similar to SSMPPLE procedure, all the port sites were infiltrated with lignocaine/bupivacaine mixture and closed by 3/0 monofilament absorbable subcuticular sutures.

### 2.13. Follow-Up Protocol

All the patients from both groups were followed meticulously every 3 months in the first postoperative year and then yearly thereafter. These patients were assessed for port-site hernias by clinical examination and ultrasound if required. However, we lost follow up to 21 patients (SSMPPLE group) and 19 patients (CMLC group).

## 3. Results 

### 3.1. The SSMPPLE Group

The mean operative time was 43.8 min (range, 20–85). The average blood loss was 9.4 mL (range, 5–55). There was no bile duct injury. However, we had one electrosurgical burn to the second part of the duodenum which was sutured by the intracorporeal technique. Eleven patients (3.4%) had small perforation of gallbladder while dissecting. Spilled bile was sucked and the stones were extracted before giving a thorough peritoneal irrigation with saline. Six patients (1.9%) had to be converted to 4-port CMLC. Five of them had intense pericholecystic adhesions not amenable to this technique and one had ambiguous biliovascular anatomy requiring conversion for better definition of critical structures. Furthermore, we converted five patients to open cholecystectomy; out of these, three were due to uncontrollable cystic artery bleeds and two were due to inadvertent gallbladder fossa bleeds requiring suturing. Eleven patients from this series had low-inserting cystic ducts, 8 had their cystic ducts opening in their right hepatic ducts and 4 had their right hepatic arteries tortuously occupying the cystohepatic triangles—the “caterpillar turns” All the patients were allowed to have solid food by 5.7 h (range, 5–12) after the surgery and were ambulatory by then. Mean VAS applied to all the patients on the days 0, 1, 7, and 30 of the surgery was 3.2 (range, 3–5), 2.1 (range, 1–4), 0, and 0, respectively. Mean postoperative analgesics were used for 1.7 days (range, 0.5–4.8). The postoperative analgesia regimen was standardized for both the groups as follows. All the patient of this study received intravenous aqueous diclofenac sodium at the end of 6th postoperative hour before putting them on oral diclofenac sodium preparation (sustained release) the next day. None of our patients needed opioid analgesics. The patients were discharged after an average of 1.3 days (range, 1–5). The mean time to take up normal activity was 3.2 days (range, 3–7) (Table 2). Except 4, all other patients are under regular follow up. While the first patient of our series has finished 4 years and 9 months of follow up, the last patient has completed 1 year and 10 months of follow up. Two of the four patents lost follow up due to their demise owing to cardiac ailments. Other two have completely lost their follow up due to the reasons unknown. Six patients (1.9%) developed umbilical sepsis which was controlled by antibiotics. Seven patients developed umbilical seroma; they recovered completely by an expectant line of treatment. None of our patients has developed trocar-site hernia till date. Seven patients (4 at the end of 9 months and 4 at the end of 13 months) developed residual bile duct stones which were extracted by endoscopic sphincterotomy. Assessment by the scar grading scale revealed 73.01% patients being thrilled and 25.56% being happy. While nobody was unhappy, 1.42% did not bother about their cosmetic outcome.

### 3.2. The CMLC Group

**Figure 5:**
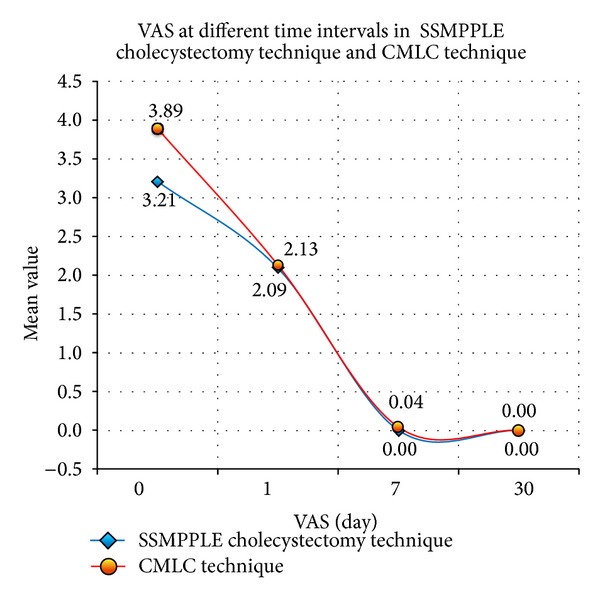
Visual Analogue Scale for the SSMPPLE cholecystectomy technique and the CMLC techniques.

In this group, the mean operative time was 39.5 min (range, 28–106) and the blood loss was 8.7 mL (range, 5–40). There were no bile duct or viscus injuries. Nine patients (2.8%) had small gallbladder perforations. Four of them had controlled stone spillages and all the stones could be “berry-picked” into the endobags. The mean VAS applied the patients on the days 0, 1, 7, and 30 of the surgery was 3.9 (range, 3–6), 2.1 (range, 2–4), 0.04 (0-1), and 0, respectively. The mean time to discharge from the hospital was 1.2 days (range, 1–7). Six patients (1.8%) developed umbilical seroma and 5 patients (1.5%) developed umbilical sepsis. All of them recovered with conservative line of management. The blood loss in SSMPPLE (9.4 mL) was significantly more than that in CMLC (8.7 mL). There were statistically significant differences in favor of SSMPPLE over CMLC as far as the operative time, VAS on postoperative days 0 and 7 (Figure 5), time for ambulation and commencing oral intake, resuming normal activities, and scar grading were concerned. We converted two patients to open cholecystectomy for cystic artery bleeds (*n* = 1) and ambiguous biliary anatomy (*n* = 1) (Table 2).

## 4. Discussion 

Owing to the obvious advantages associated with minimally invasive surgery like the less pain and the faster recovery, late 1980s saw the multiport CMLC being quickly accepted as the gold-standard for treating gallstone diseases [2–4]. Once the benefits of minimizing the access trauma, and, at the same time, having a much superior cosmetic outcomes without compromising the safety were further appreciated, the surgeons started attempting different techniques to reduce the number of ports to three or even two for laparoscopic cholecystectomy.

The late 1990s' invention—the natural orifice transluminal endoscopic surgery (NOTES)— could reduce the abdominal access trauma to zero and offered a much sought for outcome—the scarless abdomen [1, 2, 5]. Although better cosmetically, such surgeries, whether pure or hybrid, tend to have a steep learning curve owing to the complex ergonomics, the long flexible instruments with the negligible tactile feedback, and, last but not the least, the high cost factor. Not surprisingly, the transumbilical surgery, considered being the link between the conventional multiport laparoscopic surgery and the NOTES, evolved to be the most user as well as the consumer-friendly alternative. The umbilical cosmetic outcome resembled NOTES. With no risk of visceral transgression, the single-port transumbilical laparoscopic surgery was termed superior to NOTES [6, 7].

Reports discussing the feasibility of single-port transumbilical laparoscopic surgery have peaked only in the last half-a-decade with myriad of modifications [8, 9]. This might be the result of the rising demand of such surgeries producing good cosmetic results (even from the rural population like our center) coupled against the backdrop of the difficulty in learning and affording the NOTES. The single-port transumbilical laparoscopic surgery entails incising the skin and the fascia for up to 3.5 cm at the umbilicus [10, 11]. Raising the skin flap remains the unavoidable step which may contribute to the subcutaneous seroma formation and/or the skin necrosis. This potentially results in poor wound healing and inferior cosmetic results. On the contrary, the SSMPPLE eliminates this step.

We used the standard port-closure needle (coupled with catgut loop) to retract the gallbladder fundus in 46 cases of SSMPPLE. It mirrors the fourth retracting port of conventional laparoscopic cholecystectomy which allows achieving the “critical view of safety” of Strasberg and Soper [12]. Also, it helps to have the perpendicular cystic duct clipping rather than the tangential—an important step to minimize the postoperative bile leak [13]. As the gallbladder wall is not traversed by the needle, it does not violate the basic principles [13]. Further, this site can also be used for the miniscope to visualize umbilical adhesions (if any) before porting. Small drain tube can also be inserted through it, if required. However, its negligent movement can traumatize the diaphragm or the other viscera. Also, for large liver, one should avoid force retraction and opt for an additional 5 mm trocar for safe dissection. We used such an additional 5 mm trocar in the SSMPPLE group for 18 out of 46 patients.

We feel that* all *the three fascial punctures of the ports should be closed under vision. Although the cases discussed here need further long-term followup, none of our patients developed port-site herniation. Port closure under direct vision adds further to the safety.

Umbilical sepsis in the single-port transumbilical laparoscopic surgery is reported in the range of 0 to 14% [14]. We had six patients (1.9%) from the SSMPPLE group that developed umbilical sepsis; three of them were diabetic. All of them recovered completely with antibiotics. As reported earlier, we always use endobags for the gallbladder extraction [15]. This potentially reduces the umbilical contamination. The conversion rates reported in the literature are 0–24% for the single-port transumbilical laparoscopic cholecystectomy [14, 16]. In our series, it was 1.9%. However, we should keep a low threshold for conversion to standard multiport laparoscopy or open surgery [14, 17]. Furthermore, Blinman has elegantly discussed the relationship of tension (and hence pain) at the incision site to the lengths of the incision; the tension is directly proportional to the square of lengths of incisions and not the addition of the lengths [18]. Hence, the projected amount of tension acting at the three ports of SSMPPLE technique (476.1 units) would be lesser by a third than that produced at 25 mm incision of the single-incision surgery (1540.6 units).

A recent meta-analysis of 13 randomized trials (including 923 patients) that studied comparisons between single-incision laparoscopic cholecystectomy and conventional cholecystectomy reported higher failure rate, operative time, and blood loss with the former [19]. The two approaches were found comparable in terms of conversion to open surgery, length of hospital stay, postoperative pain, port-site infections, or hernias. The cosmetic outcomes were better for the former especially when 10 mm ports were used in the latter. However, we feel that, with the technical modifications described in this paper, we could achieve acceptable results. Further, we need to state at this point that the only similarity between SSMPPLE and single-incision laparoscopic cholecystectomy is the very site of access (i.e., the umbilicus). Rest all the elements in this technique (like the number, the placement and the sizes of incisions, the instruments used, the ergonomics, etc.) differ largely. Thus it tends to amalgamate the operative site (umbilicus) of the single-incision laparoscopic cholecystectomy and the instrumentation with operative techniques of the gold-standard—CMLC. Hence it should not be considered a modification of the single-incision laparoscopic cholecystectomy but should rather be taken as a distinct laparoscopic cholecystectomy technique.

A similar technique described in literature [17] used all 5 mm ports and joined the two port sites for the specimen extraction. However, we think that 10 mm laparoscope should always be used right from the commencement of the surgery as it gives much brighter, clearer, and wider vision. Also, it can be used for the 10 mm clip applier and the specimen extraction. For initial few cases of our series, the operative time was longer as our surgical team was under the learning curve of this technique. As the number of cases and the experience increased, the operative time went on decreasing. Another recently reported method uses three ports at periumbilical location to carry out cholecystectomy [20]. Although the reported technique achieved triangulation, the port placement was away from the umbilical fold. Thus, the scars did not recede within the umbilicus. The SSMPPLE helps the scars to recede at the umbilicus to produce better aesthetics.

However, the SSMPPLE has certain limitations. (i) If not precisely and strategically placed, the ports can lie too close to each other leading to extracorporeal clashing. (ii) Although it may be technically easy in wide umbilicus, a narrow or a “slit-like” umbilicus may pose a real challenge. In fact, we should keep a very low threshold for conversion to the CMLC in these cases. (iii) If the cutaneous and the fascial portal punctures lie in vertical line (rather than oblique), one may end up in having the instruments lying parallel to each other leading to difficulty in dissecting. Moreover, notable flaws of this study are (1) limited cohort, (2) nonrandomized study, (3) limited duration of the followup for drawing definitive conclusions about rate of port-site hernia, and (4) the Visual Analogue Scale for incision-related pain and the scar grading scale assessing the respective parameters in a subjective manner rather than the desired objective manner.

Although we have not conducted any cost-analysis comparisons in this study, given that the routine laparoscopic instruments were used with better operative timings without any major complications (Table 2), we feel that the SSMPPLE may become a valuable option of the per-umbilical laparoscopy especially for the patients of the developing nations. However, this technique is a modification of minimally invasive cholecystectomy. We further stress that it is* not* a modification of single incision laparoscopic cholecystectomy in any way because it includes three separate skin incisions/punctures.

## 5. Conclusion

The presented SSMPPLE cholecystectomy technique does not need any specialized ports or other equipment; it seems safe, efficient, and potentially economically viable alternative to the single-incision laparoscopic cholecystectomy using commercially available specialized port/instruments.

## Figures and Tables

**Table 1:** Patient demographics.

Patient variables	SSMPPLE	CMLC
Number of patients	320	326
Sex (male : female)	99 : 221	95 : 231
Mean age (years)		
Male	42.5 (range, 17–64)	43.8 (range, 15–67)
Female	45.3 (range, 22–68)	44.9 (range, 16–70)
Mean BMI (Kg/m^2^)		
Male	27.7 (range, 17–31.5)	25.3 (range, 18–30)
Female	28.4 (range, 19–33.7)	27.5 (range, 18–32.3)
Indications for cholecystectomy		
Biliary colic	161	160
Acute calculus cholecystitis	20	15
Chronic calculus cholecystitis	95	110
Gallbladder polyp with cholelithiasis	7	8
Mucocele of gallbladder	7	15
Empyema of gallbladder	10	4
Biliary pancreatitis	20	14
Medical comorbidities		
HTN	16	15
DM	17	19
HTN + DM	14	15
Heart disease		
Old healed MI	6	5
Left ventricular hypertrophy	5	6
Pulmonary disease		
Old healed tuberculosis	18	21
COPD (controlled)	4	7
Previous abdominal surgery (scar)		
LTL (umbilical)	22	25
LA (umbilical + right iliac fossa + suprapubic)	6	4
Laparotomy (midline)	6	5

HTN: hypertension, DM: diabetes, MI: myocardial infarction, LTL: laparoscopic tubal ligation, OA: open appendectomy, LA: laparoscopic appendectomy, SPC: suprapubic cystostomy, and DL: diagnostic laparoscopy.

**Table 2:** Results.

Perioperative variables	SSMPPLE	CMLC	*P* value
Intraoperative			
Camera assistant			
Fellow	216	168	—
Registrar	104	158	—
Mean operative time (min)	43.8 (range, 20–85)	39.5 (range, 28–106)	0.00370
Mean blood loss (mL)	9.4 (range, 5–55)	8.7 (range, 5–40)	<0.0001
Bile duct injury	0	0	—
Major vessel injury	0	0	—
Rate of conversion			
To conventional laparoscopic cholecystectomy	6	Not applicable	—
To open cholecystectomy	5	2	—
Postoperative			
Pain (mean visual analogue score)			
Day 0	3.21 ( range, 3–5)	3.89 (range, 3–6)	<0.0001
Day 1	2.09 (range, 1–4)	2.13 (range, 2–4)	NS
Day 7	0	0.04 (range, 0-1)	0.00018
Day 30	0	0	—
Mean postoperative analgesics used (days)	1.7 (range, 0.5–4.8)	3.3 (range, 1–5)	<0.0001
Ambulation (hr)	4.6 (range, 4–8)	4.8 (range, 4–12)	<0.0001
Mean time to solids after surgery (hr)	5.7 (range, 5–12)	6.6 (range, 6–12)	<0.0001
Mean time to discharge after surgery (days)	1.3 (range, 1–5)	1.2 (range, 1–7)	NS
Mean time to normal activity (days)	3.2 (range, 3–7)	3.4 (range, 3–5)	0.00444
Mean time to work (days)	9.6 (range, 7–18)	10.5 (range, 7–15)	<0.0001
Umbilical sepsis	6 (1.9%)	5 (1.5%)	NS
Umbilical seroma	7 (2.2%)	6 (1.8%)	NS
Trocar site hernia	0	0	—
Scar grade	1.28 (range, 1–3)	2.03 (range, 1–3)	<0.0001
